# Wheat Germ as Partial or Total Substitutive of Lean Meat in Low-Fat Cooked Sausages

**DOI:** 10.3390/foods14020178

**Published:** 2025-01-08

**Authors:** Marta Rodríguez-Fernández, Isabel Revilla, Pablo Rodrigo, Rocío López-Calabozo, Ana María Vivar-Quintana

**Affiliations:** Area of Food Technology, Polytechnical Superior School of Zamora, Universidad de Salamanca, Avenida Requejo 33, 49022 Zamora, Spain; martarf98@usal.es (M.R.-F.); pablo.rodrigo.dli@usal.es (P.R.); rociolc@usal.es (R.L.-C.); avivar@usal.es (A.M.V.-Q.)

**Keywords:** frankfurters, emulsion stability, nutritional characteristics, fat oxidation, sensory characteristics, consumer preference

## Abstract

Wheat germ is a byproduct of the cereal industry that contains high levels of protein, fiber, B vitamins, minerals, and other functional microcomponents. However, so far, few applications have been found in the meat industry despite the growing interest in replacing meat with vegetable proteins. Therefore, the use of wheat germ for the production of low-fat frankfurters was considered. Five different formulations were prepared: control with pork meat and the following four to achieve 25%, 50%, 75%, and 100% lean meat substitution by wheat germ. Proximal composition, color, texture, emulsion characterization, fatty acid profile, fat oxidation, and consumer acceptance were then analyzed. The results showed that the incorporation of wheat germ improved emulsion stability, decreasing significantly total expressible fluid and jelly/fat separation, although increasing the back extrusion force. In terms of the final product, the progressive substitution of meat by germ resulted in significant increases in carbohydrates, in special of fiber, and ash as well as significant decreases in moisture and total fat. Sausages made with germ were darker (L*), as well as harder, chewier, and gummier, but less cohesive and elastic. Similarly, wheat germ substitution improved the quality of the lipid profile showing higher levels of, but decreased acceptability for replacements > 25%. Substitution of meat was feasible up to 25%, a formulation for which there was hardly any significant difference with the control.

## 1. Introduction

Meat has a high content of macronutrients, mainly proteins, and micronutrients, among which vitamin B12, zinc, and iron stand out [1]; however, a probable correlation has also been postulated between the occurrence of colorectal cancer and red meat consumption [2], as well as with the development of diseases, especially cardiovascular diseases and type 2 diabetes [3]. On the other hand, it has been shown that its production is associated with a high environmental cost [4]. Thus, it is estimated that the livestock sector accounts for 15% of the anthropogenic greenhouse gases emitted globally per year [5]. Because of that, in recent years, new possibilities for the substitution of conventional animal proteins are emerging. These include insects due to their nutritional properties and low environmental impact [6] or cultured meat that would reduce the environmental impact, increase animal welfare, and reduce risks in terms of food safety [7]. However, the option that offers the widest range of possibilities is plant-based proteins [8].

Most of the meat replacements are derived from soy protein because it has desired specific traits and is available at a low cost [9]. Other pulses such as lentils, chickpea, lupine, peas, and different kinds of beans have been also studied, with pea protein being the most viable for meat-substitute applications [10,11]. Edible fungi and microalgae have been also used as textured protein sources in a meat-alternative formulation [8]. Finally, meat alternatives are also manufactured by using proteins from cereals like maize, rice, oat or wheat [12]. Therefore, oat protein isolates have been reported to produce a good sensory effect [13] and rice protein is gaining relevance by its low allergenicity [14], nonetheless, wheat proteins are the most used for plant meat based analogues [15]. Although cereals can reach values of 13–16% of protein in durum wheat or 15% in certain rice varieties [16] and show excellent water-binding properties [17], cereals contain significant amounts of phytic acid, considered anti-nutritional, while wheat contains gluten that may be avoided by people suffering celiac disease or gluten intolerance [18].

When wheat grain is processed, germ is separated during milling as a byproduct; however, wheat germ is considered the most nutritious part of wheat kernel. It contains approximately 25% protein, 18% sugars, 20% starch, and 10–15% oil [19]. The proteins (mainly albumins and globulins) have a well-balanced amino acid composition whereas cystine is lacking [20]. It is also characterized by considerable amounts of magnesium, zinc, phosphorus, and vitamin E [21,22,23]. With regard to its lipid fraction, it is characterized by being composed mainly of monounsaturated and polyunsaturated fatty acids. However, due to the presence of lipase and lipoxygenase, lipid oxidation occurs [24], producing rancidity in short periods of time, which means that the germ has a low conservation capacity and, therefore, its main use has been for animal feed [25]. However, recently, different uses of wheat germ in food have been reported, such as a fortifier in cookie production [26], in mineral-rich cakes [27], in fermented functional beverages [27], or as a coffee substitute due to its similar sensory characteristics [28].

On the other hand, the use of wheat germ in the meat industry is quite limited, finding that its oil has been used to replace animal fats in hamburgers [29], as a binder in sausages up to contents of 4% [30], or as a meat substitute up to proportions of 10% and 15% [31]. Nevertheless, there is no evidence that it has been used as the main source of protein until the partial replacement of meat in frankfurter-type sausages.

Frankfurters are a type of cooked and cured sausage [32] with a high consumption, due to their easy preparation, versatility, and organoleptic properties [33]. They are characterized by containing between 10 and 20% fat [34] with a high proportion of saturated fatty acids [35]. However, and due to the current food trends in nutrition, considerable efforts have been made in reducing the fat content [36], and to enhance the lipid profile by substitution of the fat, as reviewed by Badar et al. [37], or protein source [8]. In this scenario, proteins of vegetable origin have lower fat and cholesterol content and involve lower cost, which have favored the emergence of meat protein analogues [38,39] with the possibility of use in sausage production. In recent years, the feasibility of making frankfurters with flaxseed, coconut and oat flours [40], with textured pea protein [41], grey oyster and chickpea [42], and even macroalgae to fortify frankfurters [43] have been explored. However, as stated above, the use of wheat germ has been less investigated.

Therefore, the objective of this work was to evaluate the effect of the progressive and complete substitution of lean pork by wheat germ, as well as its viability from the point of view of emulsion stability, physicochemical quality, and sensory acceptability.

## 2. Materials and Methods

### 2.1. Food Materials and Additives

Wheat germ was supplied by the “Harina Tradicional Zamorana” Quality Label (Zamora, Spain) while pork shoulder meat, sodium chloride, and olive oil (0.4° Carbonell, Cordoba, Spain. Fatty acid composition: C16:0 11.9%, C18:0 3.8%, C18:1 71.6%, C18:1n7 2.4%, C18:2n6 6.6%) were purchased from a local market (Zamora, Spain).

Phosphates (E 451i, E 450i), potato flour, and soy protein were obtained from Proanda S. A. (Seville, Spain); sodium nitrite (E-250) and dextrose (D (+) Glucose) from Merck Eurolab (Briare Le Canal, France); and sodium lactate (E 325) and sodium ascorbate (E 301) from Panreac (Barcelona, Spain). The commercial mix of locust bean and xanthan gum (E-415, E-410, E-1400, Premigum XME-54) was supplied by Premium Ingredients S. L., (Murcia, Spain) and the vegetable sausage flavoring was supplied by Kasteel Iberoalimentaria (Toledo, Spain). Cellulose casings of 22 mm (Viscofan, Pamplona, Spain) were used for stuffing the batter.

### 2.2. Sausage Manufacture

The low-fat olive oil control cooked sausage was prepared according to the formulation proposed by Revilla et al. [41] using olive oil as the fat source. The experimental sausages were elaborated by progressively substituting the pork lean meat by wheat germen in percentages of 25% (WG25%), 50% (WB50%), 75% (WG75%), and 100% (WG100%). Therefore, five different formulations (Table 1) were manufactured in duplicate two different days with different meat and wheat germ.

Wheat germ was previously ground (HR1393 Daily Collection Phillips, Drachten, The Netherlands) to facilitate homogenization. The lean meat and the olive oil were stored at 2 °C until used.

Two products trials were elaborated according to the procedure described by Revilla et al. [41] in the pilot plant of the Area of Food Technology. A bowl cutter (Talsa T-2473, Valencia, Spain) was used, and the ingredients were added as follows: First, the soy protein and one third of the ice were mixed at low speed. Next, lean meat or wheat germ and one third of the ice were added at high speed, along with phosphate and nitrite salt. When it was completely mixed, the remaining one-third of ice and olive oil were added to obtain a homogeneous mass. Finally, sodium lactate, sodium ascorbate, dextrose, potato flour, locust bean/xanthan gum spices, and flavorings were added at low speed.

Immediately after chopping, the batter was stuffed by means of a piston stuffer (Talsa H262A, Valencia, Spain) into cellulose casings and linked at 15 cm intervals. They were then cooked in an Eller oven (Unimatic Micro model, Eller, Merano, Italy) starting with drying (15 min at 55 °C and 60% relative humidity RH), followed by heating (15 min at 60 °C and 75% RH) and finally steam cooking (75 °C until the internal temperature reached 72 °C). The cooling process included a shower until the internal temperature reached 20 °C (monitored throughout by thermocouples inserted in the thermal center) and then chilling to 4 °C overnight. Then, the frankfurters were weighed, peeled, and vacuum packed (Tecnotrip V220, Barcelona, Spain) in polyethylene bags. The sausages were pasteurized (water bath at 75 °C for 45 min) and afterwards cooled in cold running water for 15 min before being stored at 4 °C.

### 2.3. Jelly and Fat Separation

The determination of these parameters was carried out as described by Bloukas and Honikel [41]. Three pre-weighed cans (58 × 73 mm) were filled with a precisely weighed amount of raw batter, closed, and heated in a boiling water bath for 35 min (core temperature about 90 °C) and then cooled with cold tap water. After 24 h at 4 °C they were reheated to 45 °C for 1 h in a water bath. The supernatant liquid (liquid fat and gelatin) from each can was collected in a volumetric measuring cylinder and measured in ml. The separation of fat and gelatin was then calculated as a percentage of the weight of the original mass.

### 2.4. Emulsion Stability

Emulsion stability was determined as previously described by Lurueña et al. [44]. Exactly 25 g of the emulsion was weighed into centrifuge tubes (five replicates per formulation) and centrifuged for 1 min at 2958× *g* (Sigma 4K15, Osterode am Harz, Germany). The tubes were heated in a water bath (30 min at 70 °C) and centrifuged again (3 min at 2958× *g*). The pelleted part of the samples was removed and weighed, while the supernatants were dropped into crucibles previously weighed and dried at 100 °C until constant weight. The volume of the total expressible fluid (TEF) the TEF percentage (%TEF) and the fat percentage were calculated as follows:

TEF = (Weight of centrifuge tube and sample) − (Weight of centrifuge tube and pellet),(1)

% TEF = TEF/sample weight × 100(2)

%Fat = [(Weight of crucible + dried supernatant) − (Weight of empty crucible)/TEF] × 100(3)

### 2.5. Proximate Composition Analysis

Chemical analysis of the wheat germ and frankfurters was performed according to the AOAC methods [45]. Moisture was determined by oven-drying (AOAC 950.46), ash by incineration at 550 °C (AOAC 920.153), total fat by the Soxhlet method using ethylic ether (AOAC 985.15), and total protein by the Kjeldahl method using 6.25 as a conversion factor (AOAC 992.15). Starch content was determined using the enzymatic method (AOAC official method 996.11), and fiber was determined using an ANKOM analyzer (ANKOM technology, New York, NY, USA) (AOAC official method 991.43). Total carbohydrates were found by difference, using the following formula:

%Carbohydrates = (100 − Moisture − Protein − Fat − Fiber − Ash)(4)

All the analyses were performed in triplicate.

### 2.6. Texture and Color

The back extrusion method described by Hughes et al. [46] was used to determine the texture of the emulsion. Three cylindrical back extrusion vessels (50 mm of internal diameter) were carefully filled to avoid air bubbles with 100 g of emulsion immediately after chopping and tempered to 20 °C. One compression cycle was applied up to 20% of sample height at constant speed of 1 mm/s using the 40 mm compression disc and maximum compression force was recorded. Three replicates of each batch was carried out.

To determine the texture of the final product, the sausages were heated in a water bath at 70 °C for 15 min and then divided into 1-cm slices. These samples were subjected to Texture Profile Analysis (TPA) using a cylindrical probe of 50 mm diameter, a speed of 1 mm/s and a compression of 0.5 cm (50% of the sample height) [41]. Ten replicates of each sausage elaboration were analyzed. All the texture measurements (back extrusion and TPA) were carried out using a TA-XT2i texturometer (Stable Micro Systems, Surrey, UK).

Color was measured, ten replicates per formulation, on the internal surface of the longitudinally cut sausages using a HunterLab MiniScan EZ45/0 LAV colorimeter (Hunterlab, Reston, VA, USA) equipped with a 25 mm measuring head. The CIELab parameters L* (lightness), a* (redness), and b* (yellowness) were determined using a 10° observer and D_65_ illuminant.

### 2.7. Fatty Acid Profile and Fat Oxidation Stability

Intramuscular lipids were extracted using the procedure of Folch et al. [47]. The extracted fatty acids (0.1 g) were methylated with methanol: sulfuric acid (15:1) by heating in water bath at 100 °C for 1 h. After cooling, 2 mL of water and 1 mL of hexane were added and the mixture was centrifuged (3750× *g*, 5 °C, 10 min). The organic layer was taken and filtered through cotton and anhydrous NaSO_4_.

The methylated fatty acids were analyzed by gas chromatography using a GC 6890 N (Agilent Technologies, Santa Clara, CA, USA) equipped with a FID detector and using a fused silica capillary column (100 m × 0.25 mm × 0.20 µm SP-2560, Supelco, Inc, Bellefonte, PA, USA). One microliter was injected into the chromatograph in split mode (75:1) and helium 1.3 mL/min was used as carrier gas. The temperature of the injector and detector was 250 °C. The oven temperature program started at 100 °C which was maintained for 6 min, followed by 25 °C/min increases up to 200 °C, at which point it was held for 6 min. The temperature was then increased to 220 °C at 5 °C/min, followed by increases of 0.5 °C/min up to 230 °C and 4 °C/min up to 250 °C. The different fatty acids were identified by the retention time using a mixture of fatty acid standards (FAME Mix 37 components C4-C24, Supelco Inc., Bellefonte, PA, USA) to which methyl cis-7,10,13, 16-docosatetraenoate (C22:4) and methyl-all-cis-7,10,13, 16, 19-docosapentaenoate (C22:5) (Supelco Inc., Bellefonte, PA, USA) were added. The fatty acid contents were calculated using chromatogram peak areas and were expressed as mg per 100 g of fresh product. All analyses were performed in triplicate.

Fat oxidation was measured according to the method of Buege and Aust [48] to determine the thiobarbituric acid reactive substance (TBARS) content of the samples and expressed as mg of malonaldehyde (MDA) per kg of sample.

### 2.8. Sensory Analysis Evaluation

A 37-member untrained panel of regular consumers evaluated the sausages during the first week of storage using a hedonic test according to the international standard ISO 11136:2014 for consumer testing in a controlled area. Consumers were volunteers and they expressed they signed an informed consent. The test was conducted in the sensory analysis laboratory of the Food Technology area, equipped with individual booths. Samples of each formulation were pan-fried until the surface turned brown [44] and coded with a three-digit number. All consumers tasted all samples by sequential monadic presentation in random order along from 10 to 12 h. Consumers were instructed to clean their mouths with water and bread between samples to cleanse their palates. All samples were presented in the sequential monadic test using complete block design. No information about the samples was given to the consumers and they did not receive any monetary incentive for their participation to prevent bias They were also provided with a sensory card containing a 9-point structured hedonic scale ordered from least (I dislike very much) to most acceptable (I like very much). Consumers could freely and optionally express any comments related to the texture, taste and smell of the products.

### 2.9. Statistical Analysis

The significance of the replacement of meat by wheat was stablished by one-way analysis of variance (ANOVA) at an α = 0.05 level by using the F-test. The existence of statistically significant differences between samples was tested by the Tukey test, using in all the cases the SPSS Package 25 (IBM, Chicago, IL, USA).

## 3. Results and Discussion

### 3.1. Emulsion Characteristics

Results shown in the Table 2 reveal that there was a progressive increase in both force and area obtained during the back extrusion of the cold emulsion due to the incorporation of wheat germ. In the case of force these differences were significant from 75% substitution onwards, while in the case of area only for 100%. This result correlates with those previously observed by Thushan Sanjeewa et al. [49] who found that the use of pulse flour as a binder increases viscosity in raw batters.

Regarding the emulsion stability parameters, although a significant decrease in the %TEF and a significant increase in the fat released in expressible fluid were observed, in both cases this phenomenon occurred after 50% substitution. Thus, there were no differences between the control and WG25% formulation, nor between the formulations with more than 50% germ. The improvement in emulsion stability with the incorporation of wheat germ is probably due to its excellent water-holding capacity as a result of the ability of the polar groups of its polypeptides to form hydrogen bonds with water [50]. Finally, the replacement of lean pork with wheat germ also resulted in a significant reduction in fat/gelatin separation, which became less as the percentage of germ increased. However, there was no difference between the formulations incorporating germ, as the values in all formulations were very small.

Previous work [51] has described that this parameter decreases when soy protein isolates are used as an emulsifier in pork and, on the other hand, Ahmedna et al. [52] stated that solubilized wheat protein isolates have similar emulsifying properties to soy protein isolates. Proteins and starch absorb water forming gel matrices which, when heated and in the presence of meat proteins, can form a complex three-dimensional gel network involving various forces, such as van der Waals, electrostatic and hydrogen bonding forces, which traps the fine emulsified meat particles and thus improves all parameters related to emulsion stability [49].

The results of this work were in agreement with those observed by other authors who incorporated gluten in the formulations, such as Serdaroğlu and Özsümer [30] and Kamani et al. [53], who found that the %TEF decreased significantly compared to the control. In addition to this, Kamani et al. [53] reported a significant reduction in jelly/fat separation. However, for other vegetable proteins the opposite trend has been observed, such that the back extrusion force tended to decrease, while %TEF and %fat tended to increase [41], attributing this result to the fact that the pea texturized used contained neither starch nor fiber. This result highlights the relevance of these components for emulsion stability.

### 3.2. Proximate Composition

Table 3 shows that the substitution of lean pork with wheat germ resulted in a progressive decrease in moisture, which was significant from 25 to 50% substitution and from 50% to 100%. This increasing trend is also seen for total fat content, whereby there were significant differences between the control and the formulations with germ, but not between them. Both results are due to the low water and fat content of wheat germ in relation to pork (Table 3).

Regarding protein content, increasing the wheat germ percentage did not cause significant differences between the formulations, although the values tended to increase slightly. Similarly, the analysis of ash content revealed that the incorporation of up to 75% wheat germ did not lead to significant differences compared to lower substitution levels and to the control. Fiber, total carbohydrate, and starch showed a progressive and significant increase of their values as the percentage of substitution increased. The differences were significant for fiber content from 50% substitution, so that the sausages prepared only with wheat germ had four times more fiber than the control. In the case of carbohydrates, significant differences were observed between the control and WG25% and between this preparation and WG100% but not between the other batches, while all formulations were significantly different for starch content. These results were clearly due to the composition of the germ, which was characterized by a slightly higher protein and ash content, and by a noticeably higher fiber, carbohydrate, and starch content compared to pork.

The few previous works that have incorporated wheat germ have done so with up to 20% in sausage formulation, and these have also reported a significant decrease in moisture [31,54,55]. In relation to fat, the latter two works found a significant decrease in fat that they attributed to the increased dilution of the formulations, while Elbakheet et al. [31] indicated a significant increase as a consequence of the higher fat content of the germ with respect to beef. On the other hand, El Sayed et al. [55] and Elbakheet et al. [31] found that the incorporation of wheat germ led to a significant increase in protein, ash, and fiber content, which they attributed to the high amount present in wheat germ, and a decrease in carbohydrates, as these authors calculated them by difference with respect to moisture, ash, fat, and protein. Gnanasambandam and Zayas [54], on the other hand, found that the incorporation of up to 7% germ hardly modified the amount of protein.

### 3.3. Color and Texture

The substitution of lean pork meat by wheat germ significantly affected the color and texture of the cooked sausages as shown in the Table 4.

Therefore, a significant and progressive decrease in lightness (L*) was observed, which means that the sausages became significantly darker as the % of germ in the formulation increased. Along with this decrease in L*, there was also a significant decrease in a* as germ was incorporated, regardless of the percentage of substitution. As for the b* value, there was a slight increase for higher % substitution but the trend was less clear. These results may be related to the color changes that germ undergoes during cooking due to the formation of brown pigments due to non-enzymatic browning and caramelization reactions, characterized by an increase in L* values, a decrease in a* values, and the lack of a clear trend in the yellow b* color as the heating time and temperature increase [56]. To this phenomenon should be added the decrease in the formation of nitrosomyochromogen, the compound responsible for the desirable pink color of sausages [57], as the amount of meat in the formulation decreased.

The results obtained are in agreement with the previous literature. Kamani et al. [53] found that lightness significantly decreased when gluten was used and although other authors [30,54], found no significant differences, L* values also tended to be lower in germen-containing sausages. All these previous works revealed an increasing trend in b* value when comparing control vs. germ substitution, but while the works of Gnanasambandam and Zayas [54] and Serdaroğlu and Özsümer [30] also showed a decrease in a* value, although without significant differences overall, the study of Kamani et al. [53] found an increasing trend although also without significant differences between control vs. gluten substitution.

Regarding instrumental texture, no significant differences were observed between the control sausage and WG25% for almost all the parameters studied, except for chewiness, which was significantly lower for this percentage of substitution. The rest of the formulations showed increasing values, as the percentage of substitution was higher for hardness and gumminess, with the differences being statistically significant for all the formulations, while for chewiness, only WG25% showed significant lower values. As far as springiness and cohesiveness are concerned, the trend was the opposite, and the values were significantly lower the higher the percentage of germ in the formulation. The high fiber content of wheat germ could be the cause of these results, as pointed out by Garcia et al. [58] and Viuda-Martos et al. [59]. Their results highlighted that the addition of fiber to cooked sausages and mortadella significantly increased the hardness of these products due to the bonding capacity of fiber particles and because of the emulsified protein system were strengthened through the heating process [59]. This phenomenon would be related to the water binding ability and swelling properties of insoluble fibers that would influence the texture of foods [60], increasing the consistency of meat products through the formation of an insoluble three-dimensional network [61] capable of modifying rheological properties of the continuous phase of emulsions. The influence of the added fiber on hardness and chewiness would depend on the fat level [62], type, and amount of fiber [63]. Besides this, samples containing lower level of water, i.e., samples with more than 50% wheat germ substitution (Table 3), showed the highest hardness, which was also reported for sausages added with wheat germ by Gnanasambandam and Zayas [54]. This progressive decrease in moisture observed when replacing meat with wheat germ, which could be responsible for the greater crumbliness observed in the sausages with germ (Table 4) and therefore for the observed decrease in springiness and cohesiveness.

In general, there is no agreement between previous works using wheat germ or gluten, nor with the results observed in this study for instrumental texture. Thus, some works found an increasing trend for hardness, gumminess, and chewiness, as observed in this study, but also an increase for elasticity and cohesiveness [64], while other authors reported the opposite for all parameters [53] or a decrease in hardness with non-significant differences for cohesiveness [54]. This decrease in hardness and related parameters is generally observed when meat is replaced by other vegetable proteins such as pea or soy protein [41,65], which reveals the role of fiber in the formation of a stable gel network in meat emulsions.

### 3.4. Fatty Acid Profile

The analysis of the fatty acid profile allowed for the quantification of 38 individual fatty acids and their summation according to their degree of unsaturation, saturated fatty acids (SFA), monounsaturated fatty acids (MUFA), and polyunsaturated fatty acids (PUFA), along with some nutritional ratios (P/S and n6/n3), as shown in Table 5.

Minor saturated fatty acids (those found in amounts less than 1%) showed a tendency to decrease with the incorporation of wheat germ to the sausage formulation, which in the case of C6:0, C8:0, C11:0, C13:0, C15:0, C20:0, C21:0, C22:0, and C24:0 fatty acids was not significant. However, for C10:0, C12:0, C14:0, and C23:0 the decrease was progressive and statistically significant with respect to the control for the higher substitution percentages. With respect to the major saturated fatty acids, C16:0 did not show significant differences between preparations, so the fact that wheat germ is rich in this fatty acid (Table 5) did not significantly affect the preparations with meat substitution, probably due to the strong influence of the fatty acid composition of the olive oil. As regards C17:0, a significant decrease was observed, as from 50% substitution, and in C18:0, its levels decreased significantly with respect to the control, although the most important differences are those observed between the control and WG25%, results that would be correlated with the low content of these fatty acids in the wheat germ.

Hardly any of the minor unsaturated fatty acids showed statistically significant differences between formulations. However, it was observed that while C15:1, C16:1, and C18:1n9t fatty acids tended to increase with the percentage of substitution, C24:1n9 values were lower in the sausages with germ than in the control one. Finally, the incorporation of wheat germ caused a significant decrease in the major monounsaturated fatty acid, C18:1n9c because, although germ contains a high amount (Table 5), it is lower than that of pork [66] together with the great weight that the use of olive oil in processing has on the fatty acid profile [41].

The substitution of lean meat with wheat germ did not cause significant changes in the minority polyunsaturated fatty acids C18:2 n6t, C18:3 n6, C20:2 n6, C20:3 n6, C20:3 n3, C20:5n3, C20:4 n6, and C22:2 n6, and only C22:4 n3 showed a significant decrease between the control and the higher substitution percentages. In the case of the polyunsaturated fatty acids C18:2 n6 and C18:3 n3, a significant and progressive increase was observed with the percentage of substitution due to the high contents, especially of C18:2 n6, which characterized the wheat germ used.

The analysis of the different sums of fatty acids showed that SFA and MUFA levels were reduced due to the incorporation of wheat germ. While for the former, the differences were statistically significant only between the control and all the batches prepared with wheat germ, for the sum of MUFA, the differences with the control were significant from 25% substitution but WG100% showed significant differences with the other germ batches. In addition, PUFA showed a clear tendency to increase although the differences were not statistically significant.

The n3 fatty acids showed a significant increase, as the percentage of wheat germ in the formulation was higher. On the other hand, the n6 fatty acids, although they tended to increase slightly, did not show significant differences. These results are attributable to the higher PUFA content, especially n6, and lower SFA and MUFA content of wheat germ (Table 5) compared to pork [66]. With regard to the polyunsaturated/saturated fatty acid ratio, a significant and progressive increase was observed as the % of germ increased. Moreover, the n6/n3 fatty acid ratio showed a progressive decrease, the differences being statistically significant.

The positive effect of n3 fatty acids on not only cardiovascular health but also on other Western diseases such as cancer or autoimmune disorders is well known [67]. On the other hand, n6 fatty acids are the substrate for the synthesis of various pro-inflammatory molecules, so that reducing n6 intake should reduce the inflammatory potential and thus reduce the risk of coronary heart disease. However, there are also opposing claims about the role of n6 PUFA in human health, and recent studies highlight that identifying the most optimal ratio of n6 and n3 PUFA for humans would be the key factor for human nutrition [68]. Therefore, taking into account that the WHO [69] recommends a P/S ratio greater than 0.4 and an n6/n3 ratio of 4:1, the substitution of lean pork for wheat germ was nutritionally beneficial.

Other authors [28,66] also reported that the use of wheat germ, either in its natural form or in oil, in the preparation of hamburgers decreased the content of both saturated and monounsaturated fatty acids and caused an increase in polyunsaturated fatty acids; in the studies of Barros et al. [29], the differences were significant. These authors [29] also noted that n3 and n6 fatty acids tended to increase significantly, which was attributed to the high contents of linoleic acid C18:2 n6 and linolenic acid C18:3 n3, and as a result, in their work, it was found that the n6/n3 ratio decreased and the P/S ratio increased, both significantly.

### 3.5. Fat Oxidation

**Figure 1:**
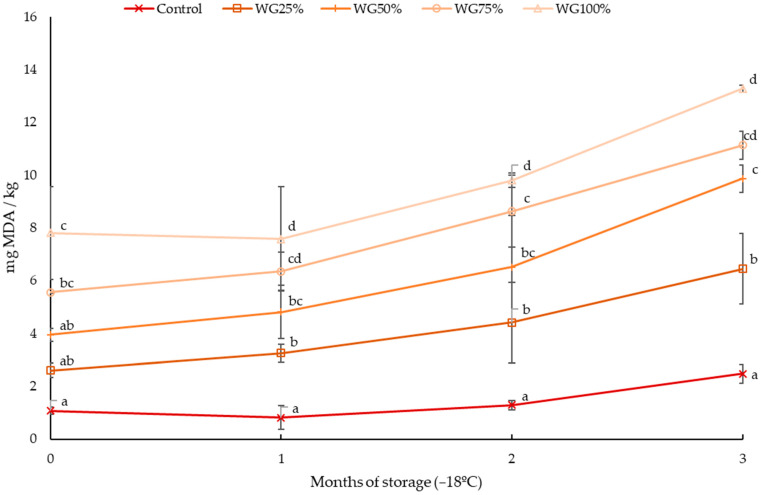
Mean values of TBARSs through the storage period of the different low-fat frankfurter formulations. a, b, c, d: different letters mean statistically significant differences between formulations at *p* < 0.05 for the same month of the storage.

In order to study the fat oxidation stability, after two days of cold storage at 4 °C, the vacuum-packed sausages were frozen (−18 °C) and stored for three months. TBARSs were determined initially and each month of the storage and expressed as mg of MDA/kg of product (Figure 1).

At the initial point of storage, the control sausages, which did not contain wheat germ, showed the lowest MDA values, which rose significantly as the concentration of wheat germ incorporated into the sausages increased, the concentrations being statistically higher from 75% substitution onwards. At this point the values obtained were above the 0.22 mg MDA/kg reported for dry pork sausages in all cases. However, the values for the control and the sausages containing up to 25% germ are close to the 1.6 mg MDA/kg reported by Coutinho de Oliveira et al. [70] for mortadella-type sausages and to the 1.06 mg MDA/kg reported by Kaczmarek et al. [71] for emulsified pork sausages. The high MDA content in wheat germ sausages is due to the fact that the germ has a high quantity of unsaturated fatty acids and high lipase (LA) and lipoxygenase (LOX) activity. In addition, the mechanical treatment used to separate the germ from the wheat kernel exposes the lipid fraction to air causing the activation of lipolytic enzymes and triggering lipid oxidation processes [72], with MDA being the main compound formed as a result of lipid oxidation in food products [73]. Unsaturated fats are hydrolyzed by the action of lipases to generate free polyunsaturated fatty acids, which are in turn substrates for lipoxygenases. The hydroperoxides resulting from the oxidative reaction are degraded into volatile and non-volatile compounds, such as alcohols, ketones, and aldehydes, which are responsible for rancid flavors and loss of nutritional properties [74,75].

During storage, a progressive increase in TBARS values was observed in all samples, being the differences statistically significant only between the starting point and the third month (*p* < 0.05) in all cases. This progressive increase in MDA values led to greater differences between formulas as storage progressed. Thus, after one month, the control sausage presented significantly lower values than the rest of the wheat germ sausages, while the WG100% formulation presented significantly higher concentrations than WG25% and WG50%. After two months, the differences were statistically significant among all batches, except WG50%, which presented intermediate values and at the third month, the WG100% and WG75% formulations presented significantly higher values than the rest of the products, which in turn were significantly different from each other. According to previous research, the kinetic profile of MDA formation in meat was divided into two stages, MDA formation was preponderant in the first stage while MDA disappearance was dominant in the second stage [76]. Thus, in the first part, the oxidation of the double bonds of unsaturated fatty acids lead to the formation of peroxides or hydroperoxides. Those that are more unstable subsequently polymerize and decompose into a wide range of compounds, including thiobarbituric acid reactive substances (TBARSs) and others such as aldehydes, hydrocarbons, ketones, alcohols, and other organic molecules [77,78]. In the second stage, MDA can react with proteins, amino acids, and phospholipids, existing in the meat [79], leading to a reduction in MDA content after a certain reaction time [80].

At the end of the storage, the absolute increase in MDA observed in wheat germ frankfurters (3.8, 5.9, 5.5, and 5.4 mg MDA/Kg for WP25%, WP50%, WP75%, and WP100%, respectively), was higher than in the control sausage (1.4 mg MDA/Kg) due to the presence of LA and LOX enzymes, which are inherent to wheat germ and which caused a higher initial oxidation, together with the higher PUFA content, which is the main substrate of these enzymes. It is noteworthy that the total increase in MDA was very similar for the higher percentages of meat substitution (>50%). The final values for control batch (2.48 mg/kg sample) were lower than those reported by Coutinho de Oliveira et al. [70], 5.6 mg/kg product for pork sausages stored for 30 days at 25 °C, and similar to those reported by Kaczmarek et al. [71] after 20 days of storage at 3 °C and Wenjiao et al. [81] after 64 days of storage at 5 °C. Sausages with germ in their composition showed higher final values. In spite of this, the WG25% batch presented final values close to those described by Coutinho de Oliveria et al. [70].

Different authors have suggested that TBARS values between 0.5 and 0.6 mg MDA/kg correspond to the detection limit for rancid odors in pork [73,77]. However, consumers carrying out the sensory analysis of these samples did not indicate in any case the existence of rancid notes, which shows that the detection limits are highly dependent on the food matrix, as previously pointed out [82].

### 3.6. Sensory Analysis

**Figure 2:**
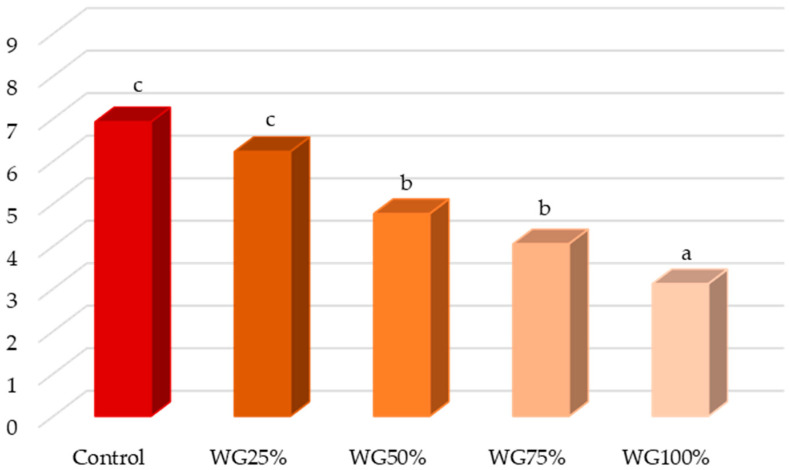
Mean values of the hedonic test of the different low-fat frankfurter formulations. a, b, c: different letters mean statistically significant differences between formulations at *p* < 0.05.

The results of the hedonic test carried out with consumers are shown in the Figure 2. It can be observed that the substitution of up to 25% of lean pork with wheat germ did not cause significant differences with respect to the control production and these batches received a rating between “I like” and “I like slightly”. The use of higher substitution percentages caused a significant decrease in the appreciation given by consumers, so that the WG50% and WG75% batches did not show significant differences between them and received a score between 5 (I neither like nor dislike) and 4 (I slightly dislike). The WG100% lot received a significantly lower score than the previous lots and its rating was equivalent to “dislike”. For this batch of sausages, consumers commented that it was too tough and had a distinctly vegetal flavor and aroma reminiscent of straw.

The incorporation of wheat germ in substitution of fat [64] or gluten in substitution of meat [53] in proportions lower than 20% did not cause significant differences with respect to the control in the overall acceptability of consumers agreeing; therefore, with the present work, pointing out that substitution in small proportions does not vary the acceptability with respect to the control. In fact, Elbakheet et al. [31], observed that substitution up to 15% of beef with wheat germ improved the overall sensory acceptability assessed by flavor, aroma, meat flavor variation, juiciness, and tenderness.

## 4. Conclusions

The progressive substitution of lean pork for wheat germ led to a progressive increase in emulsion stability, which was reflected in a lower jelly/fat separation and a lower total expressible fluid of the batter containing a progressively higher fat content. In addition, a progressive increase in batter viscosity was also observed, which produced higher values of back extrusion force. As for the cooked sausages, the results revealed that the higher the percentage of meat substitution, the lower the moisture and fat content and the higher the percentage of ash and fiber and the higher the PUFA content, both n6 and n3 fatty acids. However, although nutritional and emulsion stability characteristics improved, fat oxidation during storage was greater as the percentages of wheat germ included in the formula increased and textural properties were excessively altered. As a result, the sausages were progressively harder, chewier, and gummier, but also less cohesive and elastic, as well as darker, leading to a significant decrease in consumer acceptance.

However, the results always pointed out that the substitution of meat with 25% wheat germ in the frankfurters formulation did not produce significant changes with respect to the control in color, texture or initial MDA content, resulting in non-significant differences for consumer acceptance of this batch. Taking into account these results, future work could consider the preparation of vegetable cooked sausages combining wheat germ with other proteins of vegetable origin, in order to achieve textures as similar as possible to those of conventional cooked meat products.

## Figures and Tables

**Table 1:** Low-fat frankfurter formulations made with olive oil and different levels of meat replacement by wheat germ as a percentage of total weight.

Ingredients	Control	WG25%	WG50%	WG75%	WG100%
Lean pork	40	30	20	10	0
Wheat germ	0	10	20	30	40
Olive oil	12.5	12.5	12.5	12.5	12.5
Locust bean/Xanthan gum	0.8	0.8	0.8	0.8	0.8
Ice	36	36	36	36	36
Polyphosphate	0.3	0.3	0.3	0.3	0.3
Nitrite salt ^1^	1.6	1.6	1.6	1.6	1.6
Potato starch	2.5	2.5	2.5	2.5	2.5
Soy protein	2	2	2	2	2
Sodium ascorbate	0.05	0.05	0.05	0.05	0.05
Dextrose	0.25	0.25	0.25	0.25	0.25
Sodium lactate	1	1	1	1	1
Flavorings	2	2	2	2	2
Onion	0.55	0.55	0.55	0.55	0.55
Garlic	0.4	0.4	0.4	0.4	0.4
Pepper	0.05	0.05	0.05	0.05	0.05

^1^ NaCl + 0.6% sodium nitrite.

**Table 2:** Mean values (+SD) of emulsion texture and emulsion stability of the different low-fat frankfurter formulations.

	Control	WG25%	WG50%	WG75%	WG100%	*p*-Value
Back extrusion force (N)	3.80 ± 0.78 ^a^	5.56 ± 0.59 ^a^	9.61 ± 2.24 ^a,b^	17.48 ± 7.20 ^b^	32.18 ± 15.25 ^c^	0.000
Back extrusion area (N·s)	28.70 ± 5.67 ^a^	20.82 ± 12.13 ^a^	21.55 ± 12.76 ^a^	67.79 ± 43.74 ^a^	134.97 ± 89.16 ^b^	0.001
%TEF	4.14 ± 0.75 ^b^	3.92 ± 1.53 ^b^	2.55 ± 0.81 ^a^	1.24 ± 1.27 ^a^	1.41 ± 0.54 ^a^	0.000
%Fat	2.92 ± 1.68 ^a^	3.27 ± 0.99 ^a^	6.32 ± 2.14 ^b^	6.51 ± 0.62 ^b^	6.56 ± 0.67 ^b^	0.000
Jelly/fat separation (%)	0.193 ± 0.13 ^b^	0.074 ± 0.07 ^a^	0.013 ± 0.01 ^a^	0.005 ± 0.01 ^a^	0.003 ± 0.01 ^a^	0.000

^a,b,c^ Different letters mean statistically significant differences at *p* < 0.05.

**Table 3:** Mean values (+SD) of the chemical composition parameters of the wheat germen and of the different low-fat frankfurter formulations.

	Wheat Germ	Control	WG25%	WG50%	WG75%	WG100%	*p*-Value
Moisture (%)	8.37 ± 0.18	61.88 ± 3.20 ^c^	56.86 ± 4.20 ^c^	48.67 ± 2.59 ^b^	45.47 ± 3.94 ^a,b^	39.87 ± 5.98 ^a^	0.000
Total fat (%)	7.64 ± 0.13	12.26 ± 1.53 ^b^	9.21 ± 1.43 ^a^	8.96 ± 1.12 ^a^	8.09 ± 0.85 ^a^	7.92 ± 1.00 ^a^	0.000
Protein (%)	25.75 ± 0.21	11.51 ± 0.84 ^a^	10.90 ± 1.15 ^a^	12.20 ± 0.95 ^a^	12.11 ± 3.26 ^a^	13.27 ± 1.34 ^a^	0.226
Ash (%)	4.30 ± 0.03	3.29 ± 0.35 ^a^	3.39 ± 0.35 ^a^	3.60 ± 0.16 ^a^	3.93 ± 0.18 ^a^	4.70 ± 0.68 ^b^	0.001
Fiber (%)	24.90 ± 0.85	2.23 ± 0.95 ^a^	3.70 ± 0.29 ^a^	6.25 ± 1.67 ^b^	7.45 ± 1.65 ^b,c^	9.63 ± 1.64 ^c^	0.000
Carbohydrate (%)	29.05 ± 0.92	8.53 ± 0.27 ^a^	15.94 ± 1.66 ^b^	20.07 ± 1.11 ^bc^	21.96 ± 2.78 ^b,c^	24.61 ± 3.56 ^c^	0.005
Starch (%)	17.45 ± 0.35	3.29 ± 0.37 ^a^	5.04 ± 0.24 ^b^	6.68 ± 0.02 ^c^	7.75 ± 0.63 ^d^	9.89 ± 0.15 ^e^	0.000

^a,b,c,d,e^ Different letters mean statistically significant differences at *p* < 0.05 among the different sausages.

**Table 4:** Mean values (+SD) of instrumental color and texture parameters of the different low-fat frankfurter formulations.

	Control	WG25%	WG50%	WG75%	WG100%	*p*-Value
L*	64.24 ± 1.65 ^e^	59.04 ± 1.39 ^d^	56.82 ± 1.37 ^c^	51.10 ± 0.71 ^b^	45.50 ± 1.50 ^a^	0.000
a*	19.50 ± 0.38 ^b^	15.92 ± 0.69 ^a^	15.94 ± 0.80 ^a^	16.03 ± 1.93 ^a^	15.20 ± 1.39 ^a^	0.000
b*	30.90 ± 1.25 ^a,b^	22.88 ± 2.68 ^a^	31.07 ± 1.88 ^a,b^	33.51 ± 5.85 ^b^	31.39 ± 5.69 ^a,b^	0.069
Hardness (g)	1964.63 ± 509.48 ^a^	1901.98 ± 264.14 ^a^	2947.78 ± 556.36 ^b^	3698.22 ± 370.19 ^c^	4947.98 ± 334.02 ^d^	0.000
Adhesiveness (g·mm)	−1.32 ± 1.83 ^a^	−0.43 ± 0.79 ^a^	−0.88 ± 1.42 ^a^	−0.11 ± 0.08 ^a^	−0.30 ± 0.57 ^a^	0.682
Springiness (mm)	0.95 ± 0.15 ^d^	0.86 ± 0.05 ^c^	0.80 ± 0.05 ^c^	0.70 ± 0.07 ^b^	0.62 ± 0.40 ^a^	0.000
Cohesiveness	0.76 ± 0.04 ^d^	0.68 ± 0.05 ^c^	0.64 ± 0.09 ^b^	0.62 ± 0.06 ^b^	0.53 ± 0.05 ^a^	0.000
Gumminess (g)	1478.49 ± 315.29 ^a^	1297.29 ± 186.77 ^a^	1887.94 ± 508.74 ^b^	2277.53 ± 181.36 ^c^	2624.37 ± 210.53 ^d^	0.000
Chewiness (g·mm)	1426.65 ± 535.57 ^b^	1121.46 ± 203.04 ^a^	1532.16 ± 487.62 ^b^	1611.54 ± 269.10 ^b^	1622.40 ± 135.70 ^b^	0.000

^a,b,c,d,e^ Different letters mean statistically significant differences at *p* < 0.05.

**Table 5:** Mean values (+SD) of individual fatty acids of the wheat germ and of the different low-fat frankfurter formulations.

	Wheat Germ	Control	WG25%	WG50%	WG75%	WG100%	*p*-Value
C6:0	14.96 ± 1.54	5.22 ± 3.1	2.35 ± 3.22	10.44 ± 5.30	2.81 ± 4.01	5.08 ± 1.34	0.433
C8:0	21.14 ± 2.00	0.90 ± 0.79	n.d.	n.d.	1.18 ± 0.42	n.d.	0.318
C10:0	0.68 ± 0.15	5.36 ^b^ ± 1.94	3.92 ^a,b^ ± 1.11	4.31 ^a,b^ ± 1.05	2.61 ^a,b^ ± 0.25	1.78 ^a^ ± 0.27	0.014
C11:0	0.80 ± 0.10	n.d	n.d	8.21 ± 1.21	n.d	0.86 ± 0.10	0.267
C12:0	0.96 ± 0.51	5.10 ^b^ ± 1.79	4.04 ^a,b^ ± 1.21	3.74 ^a,b^ ± 0.85	2.36 ^a,b^ ± 0.53	1.81 ^a^ ± 0.12	0.015
C13:0	1.51 ± 0.07	9.94 ± 4.99	2.36 ± 0.42	7.77 ± 3.77	2.39 ± 1.32	0.54 ± 0.44	0.648
C14:0	7.19 ± 0.46	53.90 ^c^ ± 12.30	37.14 ^b^ ± 12.16	28.33 ^a,b^ ± 3.00	18.49 ^a^ ± 2.55	11.21 ^a^ ± 0.95	0.003
C14:1n5	1.08 ± 0.54	4.27 ± 1.30	2.82 ± 0.56	3.63 ± 1.39	3.23 ± 0.40	1.29 ± 0.23	0.126
C15:0	3.83 ± 0.23	5.41 ± 1.11	1.64 ± 1.32	2.78 ± 1.18	2.02 ± 0.48	2.34 ± 1.06	0.074
C15:1	0.65 ± 0.92	10.41 ± 3.90	18.77 ± 0.45	17.15 ± 3.33	15.82 ± 3.15	15.97 ± 3.74	0.797
C16:0	995.55 ± 2.31	2676.53 ± 176.70	2115.01 ± 157.75	2057.07 ± 87.73	1990.97 ± 151.52	1660.24 ± 138.83	0.243
C16:1	9.12 ± 0.17	33.24 ± 6.97	25.16 ± 6.80	21.18 ± 2.35	85.44 ± 9.18	84.24 ± 6.11	0.405
C17:0	10.23 ± 0.42	246.36 ^c^ ± 67.13	187.49 ^b^ ± 12.93	163.02 ^b^ ± 8.68	10.21 ^a^ ± 2.80	3.22 ^a^ ± 0.28	0.000
C17:1	1.31 ± 0.08	32.92 ± 10.37	29.61 ± 1.63	25.42 ± 2.51	25.48 ± 6.31	17.64 ± 1.37	0.170
C18:0	1.61 ± 0.11	852.51 ^d^ ± 157.12	3.20 ^a^ ± 0.61	159.68 ^b^ ± 70.10	433.10 ^c^ ± 169.12	190.74 ^b^ ± 68.48	0.006
C18:1n9t	7.75 ± 0.52	66.85 ± 13.33	82.50 ± 11.75	75.79 ± 8.02	75.60 ± 11.37	61.13 ± 5.92	0.801
C18:1n9c	607.34 ± 6.80	10632.17 ^d^ ± 976.65	8265.09 ^c^ ± 640.41	7823.80 ^b,c^ ± 102.05	7443.37 ^b^ ± 160.31	5970.93 ^a^ ± 149.21	0.041
C18:2n6t	2.59 ± 0.17	5.23 ± 1.72	11.05 ± 3.24	4.08 ± 1.69	2.41 ± 0.57	2.05 ± 0.83	0.265
C18:2 n6	631.43 ± 19.85	1800.35 ^a,b^ ± 157.53	1730.40 ^a^ ± 104.29	1943.42 ^b^ ± 107.54	2272.04 ^b,c^ ± 195.27	2053.26 ^b^ ± 194.59	0.050
C20:0	6.28 ± 6.67	73.37 ± 16.49	56.71 ± 1.57	56.38 ± 0.82	54.75 ± 5.11	43.67 ± 1.95	0.214
C18:3 n6	8.79 ± 0.60	7.95 ± 1.57	6.85 ± 0.82	9.49 ± 2.11	6.70 ± 1.95	6.21 ± 0.73	0.553
C20:1 n9	23.25 ± 0.87	75.27 ± 8.22	64.54 ± 5.00	68.97 ± 10.56	67.88 ± 8.67	59.28 ± 6.27	0.781
C18:3 n3	59.31 ± 26.72	124.39 ^a^ ± 16.88	144.42 ^a^ ± 16.77	194.20 ^a,b^ ± 13.90	257.27 ^b^ ± 28.25	247.16 ^b^ ± 28.72	0.001
C21:0	7.85 ± 0.55	4.48 ± 1.57	4.78 ± 1.82	4.53 ± 1.16	2.15 ± 0.81	3.60 ± 1.62	0.371
C20:2 n6	7.94 ± 0.31	56.44 ± 11.21	59.29 ± 6.37	33.47 ± 11.91	33.22 ± 8.59	36.55 ± 10.14	0.262
C22:0	7.19 ± 1.56	23.57 ± 4.10	20.92 ± 1.48	4.12 ± 1.94	11.49 ± 3.28	8.75 ± 2.62	0.051
C20:3 n6	1.93 ± 0.25	8.26 ± 3.54	8.88 ± 4.45	4.72 ± 1.57	3.52 ± 1.85	20.54 ± 10.09	0.451
C22:1 n9	18.21 ± 0.97	37.45 ± 10.67	42.44 ± 3.25	24.63 ± 6.33	31.71 ± 7.11	34.99 ± 10.56	0.716
C20:3 n3	3.32 ± 0.19	3.79 ± 0.77	3.59 ± 0.70	2.52 ± 0.32	12.69 ± 6.59	2.00 ± 1.73	0.371
C23:0	1.79 ± 0.05	46.97 ^d^ ± 13.16	28.76 ^c^ ± 0.98	15.46 ^b^ ± 3.97	13.88 ^b^ ± 3.35	0.47 ^a^ ± 0.82	0.000
C20:4 n6	7.43 ± 0.28	11.15 ± 3.10	13.30 ± 0.05	13.92 ± 3.07	10.29 ± 1.03	13.76 ± 2.76	0.964
C22:2 n6	4.50 ± 0.42	6.79 ± 1.19	1.17 ± 1.66	1.53 ± 1.34	2.14 ± 0.30	2.50 ± 0.52	0.715
C24:0	8.43 ± 6.65	n.d	n.d.	5.94 ± 2.26	3.72 ± 1.28	n.d.	0.468
C20:5 n3	7.89 ± 0.15	9.15 ± 2.72	8.57 ± 1.11	4.62 ± 2.43	8.21 ± 2.79	7.86 ± 1.67	0.475
C24:1 n9	14.05 ± 6.90	18.78 ± 10.42	4.89 ± 2.21	4.53 ± 0.22	5.90 ± 1.05	5.28 ± 0.38	0.705
C22:4 n3	6.60 ± 0.42	12.47 ^b^ ± 2.1	8.27 ^a,b^ ± 1.13	4.14 ^a^ ± 0.46	3.24 ^a^ ± 1.35	2.51 ^a^ ± 0.51	0.001
C22:5 n3	10.43 ± 1.21	29.54 ± 4.76	7.39 ± 1.65	22.14 ± 4.97	3.33 ± 0.85	2.27 ± 0.13	0.464
C22:6 n3	8.91 ± 7.99	n.d	n.d.	n.d.	n.d.	5.69 ± 0.86	0.397
SFA	1089.99 ± 21.06	4009.63 ^b^ ± 284.87	2472.06 ^a^ ± 199.56	2531.79 ^a^ ± 164.90	2552.14 ^a^ ± 132.65	1934.30 ^a^ ± 78.26	0.050
MUFA	711.38 ± 6.37	10905.05 ^c^ ± 495.25	8539.21 ^b^ ± 444.24	8064.70 ^b^ ± 210.10	7751.77 ^b^ ± 271.76	6247.99 ^a^ ± 267.74	0.005
PUFA	732.47 ± 49.24	1981.82 ± 271.09	1999.80 ± 146.07	2338.65 ± 157.68	2617.71 ± 132.80	2405.14 ± 173.66	0.052
n6	34.46 ± 6.26	1941.42 ± 252.30	1920.86 ± 234.40	2197.81 ± 151.51	2405.79 ± 110.82	2194.85 ± 125.46	0.810
n3	702.56 ± 26.76	137.34 ^a^ ± 53.32	156.57 ^a^ ± 19.57	201.34 ^a,b^ ± 10.79	278.17 ^b^ ± 19.45	257.02 ^b^ ± 10.92	0.001
P/S	0.67 ± 0.05	0.55 ^a^ ± 0.02	0.81 ^b^ ± 0.01	0.93 ^b^ ± 0.14	1.03 ^b,c^ ± 0.08	1.25 ^c^ ± 0.21	0.000
n6/n3	29.08 ± 2.39	13.81 ^c^ ± 0.78	12.27 ^c^ ± 0.04	10.90 ^b^ ± 0.92	8.65 ^a^ ± 0.58	8.55 ^a^ ± 0.16	0.000

^a,b,c,d^ Different letters mean statistically significant differences at *p* < 0.05 among the different sausages. n.d.: Not detected. P/S: ratio between polyunsaturated and saturated fatty acids. n6/n3: ratio between polyunsaturated n6 fatty acids and polyunsaturated n3 fatty acids.

## Data Availability

The original contributions presented in the study are included in the article, further inquiries can be directed to the corresponding author.
